# Novel organic–inorganic hybrid powder SrGa_12_O_19_:Mn^2+^–ethyl cellulose for efficient latent fingerprint recognition *via* time-gated fluorescence†

**DOI:** 10.1039/d0ra00138d

**Published:** 2020-02-26

**Authors:** Jun'an Lai, Zhangwen Long, Jianbei Qiu, Dacheng Zhou, Qi Wang, Yong Yang, Songhan Hu, Zhe Wang, Ke Zhang

**Affiliations:** College of Materials Science and Engineering, Kunming University of Science and Technology Kunming 650093 China qiu@kmust.edu.cn 363270387@qq.com +86-875-5188856 +86-871-5188856; Key Lab. of Advanced Materials of Yunnan Province Kunming 650093 China

## Abstract

Latent fingerprints (LFPs) are important evidence in crime scenes and forensic investigations, but they are invisible to the naked eye. In this work, a novel fluorescent probe was developed by integrating a narrow-band-emitting green afterglow phosphor, SrGa_12_O_19_:Mn^2+^ (SGO:Mn), and ethyl cellulose (EC) for the efficient visualization of LFPs. The hydrophobic interactions between the powder and lipid-rich LFPs made the ridge structures more defined and easily identifiable. The background fluorescence of the substrates was completely avoided because of the time-gated fluorescence of the afterglow phosphor. All the three levels of LFP degrees were clearly imaged due to the high sensitivity. Moreover, the SGO:Mn–EC powder was highly stable in neutral, acidic, and alkaline environments. In addition, 60 day-aged LFPs were successfully visualized by the powder. All performances showed that this strategy for LFP recognition has merits such as low cost, non-destructive nature, reliability, superior universality, and legible details. Together, these results show the great application prospects of this powder in forensic identification and criminal investigation.

## Abstract 2

A easy and efficient strategy for latent fingerprints recognition was developed by this work.

## Graphical abstract

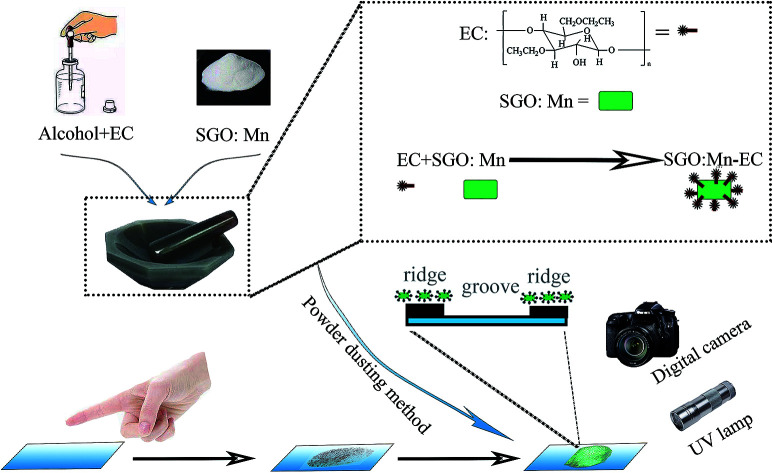

## 1 Introduction

Fingerprint recognition is the most reliable method to identify a person because of the unique characteristics of each fingerprint.^1–3^ There is an urgent need for an efficient and easy fingerprint identification method in many fields such as forensic investigation,^4,5^ law enforcement,^5,6^ and medical diagnostics.^7^ Generally, latent fingerprints (LFPs) can be visualized by creating a strong optical contrast between the surface of the fingerprint ridges and the fingerprint grooves. Due to its efficiency and simplicity, the powder dusting method is the most commonly used LFP imaging method, but its low resolution and low contrast limit its effectiveness.^5,8,9^ The photoluminescence detection of LFPs is considered to be a more effective approach due to its higher selectivity and contrast than traditional methods such as iodine fuming and vacuum metal deposition.^10,11^ Many photoluminescent probe candidates have been reported for LFP imaging, such as conjugated polymer nanoparticles,^12,13^ nanophosphors,^14^ organic aggregation-induced emission fluorophores,^4,15^ noble metal nanoclusters,^16–18^ semiconductor quantum dots,^8,19^ and rare earth upconversion fluorescent nanomaterials.^20,21^

However, these materials suffer from some shortcomings; for example, using noble metals increases the cost of the photoluminescent probes. Additionally, the emission color varies depending on the substance that is used, leading to a complex imaging process, and this method is unable to completely eliminate background fluorescence.^22^ Physically adsorbed materials have a weak affinity for ridges.^23^ Some probes can only resolve first- and second-level fingerprint features of the fingerprint degree but not the third-level features.^24,25^ Moreover, many probes reported in the literature require post processing, such as heating, surfactants, or wet chemical reactions, which may damage the appearance and structure of LFPs.^17,26^ Worst of all, the presence of background interference may reduce the intensity and contrast of LFP images, which can cause the obtained images to be seriously blurred or even completely blanked.^7,9,14,21,27^

Although these are difficult challenges to overcome, it is worth studying these materials due to their potential benefits. Previous research has reported the benefits of using phosphors for LFP imaging, which include exceptional photochemical stability, almost no morphological variations, low costs, and low toxicity.^10,28^ Background fluorescence can be completely eliminated by using phosphors with long persistent luminescence (LPL), such as those used in biological imaging, which have remarkable signal-to-noise ratios.^29,30^ The use of nano-long afterglow particles for achieving latent fingerprint visualization without background fluorescence has been reported.^31–33^ However, as mentioned above, the use of solvents during operation can damage the fingerprint topography, resulting in a decrease in the fingerprint resolution. A non-destructive and non-background fluorescence interference latent fingerprint method is urgently needed. It is important to get the most accurate fingerprint image in a short time with a simple method at the crime scene. A combination of multiple strategies could be improve the visualization of fingerprint images. Monochromatic emission of fluorescent probes can enhance the contrast of LFPs images.^14,34^ The resolution and contrast of imaging can be enhanced by using organic matter such as coumarin A with functional groups that generate electrostatic interactions to adhere the LFPs on the surface of the fluorescent powder.^4,5,8,22^

Herein, we successfully designed and synthesized a LFP probe by using a simple organic–inorganic hybrid strategy to combine the inorganic afterglow phosphor SrGa_12_O_19_:Mn^2+^ (SGO:Mn) and ethyl cellulose (EC). SGO is a superior afterglow host lattice, as reported in our previous works, and the proper crystal field environment for Mn^2+^ ions' narrow band emitting can be provided by the SGO host.^35^ EC is a common cellulose with chemical stability, thermal stability, superior cohesiveness, and desirable hydrophobicity. A series of SGO:Mn phosphors functionalized with methyl and ethyl groups in EC generated hydrophobic interactions with the LFPs. High-resolution, high-sensitivity, and high-contrast visualization were achieved due to the efficient combination of fluorescence imaging and powder dusting methods. The ion occupancy, phase, structure, luminescence, thermoluminescence, and afterglow properties of the SGO:Mn phosphor are discussed in detail. The hydrophobic interactions between SGO:Mn–EC and LFPs caused high affinity between the two materials, which performed better than mere physical adsorption. LFPs on various types of substrates could be clearly visualized. The details of LFPs are discussed and all the three levels of LFP features were clearly resolved, demonstrating the high resolution and efficiency of SGO:Mn–EC for LFP visualization. Moreover, SGO:Mn–EC could visualize aged LFPs, which is difficult to accomplish with other techniques. Thus, a technologically simple, stable, low-cost, non-destructive, sensitive, and rapid powder was developed for LFP recognition. The outstanding abilities of SGO:Mn–EC show that this material has promising practical application prospects.

## 2 Experimental section

### 2.1 Materials and synthesis

A series of SGO:*x*Mn (*x* = 0%, 0.5%, 1%, 2%, 3%, 4%, and 5%) phosphors was synthesized by a high-temperature solid-state reaction. The reagents SrCO_3_ (99.9%), Ga_2_O_3_ (99.9%), and MnO_2_ (99.9%) were weighed in a non-stoichiometric ratio due to the volatility of Ga_2_O_3_ under a reducing atmosphere. The mixed powders were ground in an agate mortar for 30 min, then transferred to an aluminum crucible, sintered at 1350 °C for 4 h in air, and then sintered at 800 °C for 8 h under a 95% N_2_/5% H_2_ atmosphere. The samples were cooled to room temperature in the furnace and then ground into powder for further use. The adopted EC (CAS no. 9004-57-3) was in white powder form. All the raw materials were purchased from Shanghai Aladdin Bio-Chem Technology (China).

**Scheme 1:**
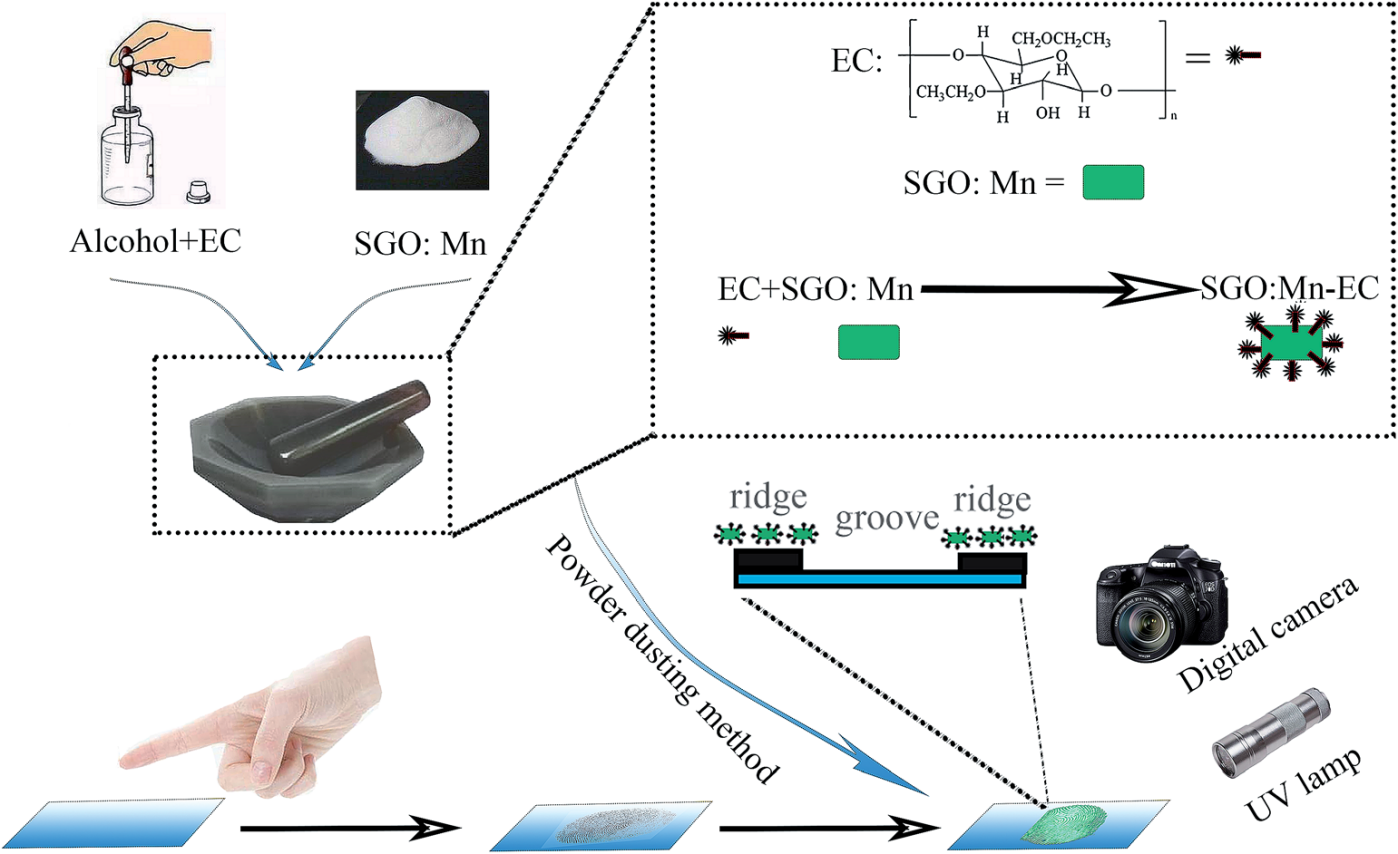
The synthesis of SGO:Mn–EC and LFP detection through the powder dusting method.

The process of preparation of the SGO:Mn–EC powder is shown in Scheme 1. First, EC was dissolved in alcohol in the ratio of 1 : 2 by mass. The solution was stirred until it was free of precipitate. Next, the solution and the previously obtained powder were added into an agate mortar in the ratio of 5% EC/95% SGO:Mn by mass. Then, the two were ground carefully until the alcohol evaporated and the components were evenly distributed. Finally, the SGO:Mn–EC mixture was obtained.

### 2.2 Characterization

The phases of the phosphors were identified by X-ray diffraction (XRD, D8 ADVANCE, Germany, Bruker), using Cu K_α_ at a scanning step of 0.02° in the 2*θ* range from 10° to 80°. The XRD data was refined by the Rietveld method using GSAS refinement program. A Hitachi F-7000 fluorescence spectrophotometer was used to record the photoluminescence (PL), temperature-dependent photoluminescence, photoluminescence excitation (PLE), and long persistent luminescence (LPL) spectra. An identical weight (2 mg) of the samples was placed into an aluminum crucible, then the thermoluminescence (TL) curves were measured on an FJ-427A TL meter (Beijing Nuclear Instrument Factory). The samples were heated from room temperature to 650 K with the heating rate of 60 K min^−1^. Afterwards, the samples were exposed to 254 nm UV light for 10 min. Fourier-transform infrared (FTIR) spectra were recorded on a PerkinElmer spectrometer (PerkinElmer, USA) using KBr pellets. The morphology and size of SGO:Mn–EC were characterized using scanning electron microscopy (SEM) images obtained using FE-SEM (FEI QUANTA-200).

### 2.3 Latent fingerprint development

LFP samples were taken from the same male donor and were prepared by lightly rubbing the finger on the nose and forehead before touching the surface of different substrates. The different substrate materials included a rough desktop, a smooth glass surface, and an acrylic sheet with variable background fluorescence. The powder dusting method was used to develop the latent fingerprints by blowing air to remove the excess powder. The photographs of the latent fingerprints were taken with a Canon 70D digital camera.

## 3 Results and discussion

**Fig. 1:**
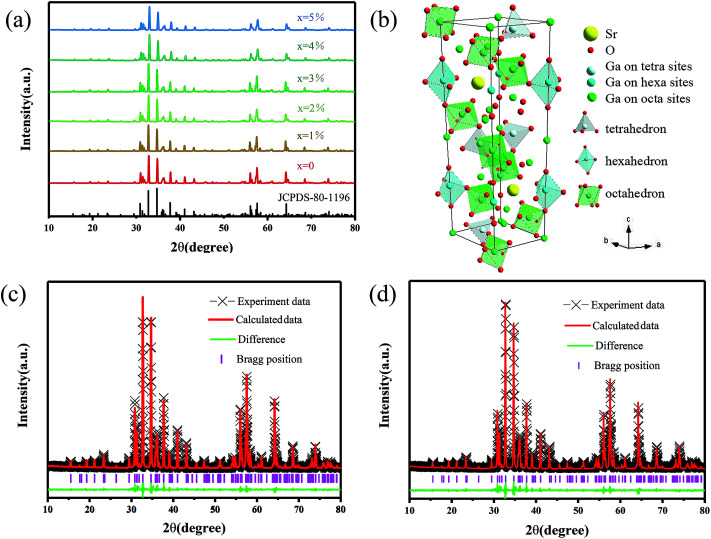
(a) The XRD patterns of SGO:*x*Mn^2+^ (*x* = 0%, 0.5%, 1%, 2%, 3%, 4%, and 5%), the standard SrGa_12_O_19_ data (JCPDS no. 80-1196) is shown as the reference; (b) the crystal structure of the SGO host, and the coordination polyhedrons of the tetrahedron, hexahedron, and octahedron of Ga coordination; (c) the representative Rietveld refinement of SGO:2% Mn^2+^ sample before annealing in a reducing atmosphere; (d) the representative Rietveld refinement of SGO:2% Mn^2+^ sample after annealing in a reducing atmosphere.

All the XRD patterns of different Mn^2+^ doped SGO phosphors closely matched the standard JCPDS card and no impurities were observed. Fig. 1(a) shows the XRD spectrum of SGO:*x*Mn^2+^ (*x* = 0%, 0.5%, 1%, 2%, 3%, 4%, and 5%) and standard JCPDS card no. 80-1196.^36^Fig. 1(b) shows the crystal structure of SGO. The ionic radii of Mn^2+^ in different tetrahedral, octahedral, and dodecahedral sites are 0.57 Å, 0.83 Å, and 0.96 Å,^37^ respectively, and the ionic radii of Ga^3+^ are 0.47 Å (CN = 4) and 0.62 Å (CN = 6).^38^ Mn^2+^ ions were substituted at the tetrahedral and octahedral Ga^3+^ sites due to their similar ionic radii.

Fig. 1(c and d) show the Rietveld refinements of the XRD patterns of a representative undoped SGO sample according to the literature pattern of the SGO reagent. The refined structural parameters are listed in Tables S1 and S2,† and the SGO cell parameters are listed in Table 1. According to the refinement data, the SGO sample crystallized in a hexagonal phase, with the space group *P*6_3_/*mmc*, *α* = *β* = 90°, *γ* = 120°, and *Z* = 2. All the atom positions, fraction factors, and thermal vibration parameters were refined by convergence and satisfied the reflection conditions. The refinement data of SGO:Mn before and after annealing under a reducing atmosphere were similar, indicating that annealing did not affect the crystallinity.

**Table 1:** Rietveld refinement data of the SGO:2% Mn^2+^ sample before (A) and after (B) annealing in a reducing atmosphere

Formula	Crystal system	Space group	*a*	*c*	*V*	*R*_wp_	*R*_p_	*χ* ^2^
A	Hexagonal	*P*6_3_/*mmc*	5.7982	22.8359	664.86	12.85	8.55	1.4821
B	Hexagonal	*P*6_3_/*mmc*	5.7982	22.8362	664.87	13.13	8.88	1.6179

**Fig. 2:**
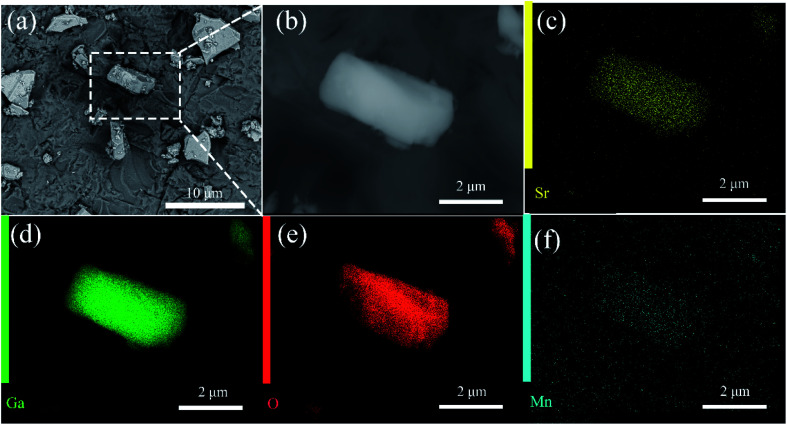
(a) SEM image of SGO:5% Mn^2+^ microcrystal particles; (b) an enlarged particle; (c–f) elemental mapping images of Sr, Ga, O, and Mn for the selected SGO:5% Mn^2+^ particle.

The scanning electron microscopy (SEM) images of SGO:5% Mn^2+^ in Fig. 2 show a particle size range of 5–10 μm with fine crystallinity. Moreover, the energy dispersive X-ray spectroscopy (EDS) elemental maps were used to confirm the compositional uniformity of SGO:Mn (Fig. 2(c–f)). According to SEM-EDS, the mass and atomic percentage of each element in SGO:5% Mn^2+^ are shown in Table S3.† Fig. S1† shows the SEM-EDS analysis of SGO:5% Mn^2+^, which confirmed the uniformity of spray-deposited Sr, Ga, and O within the phosphor particles.

**Fig. 3:**
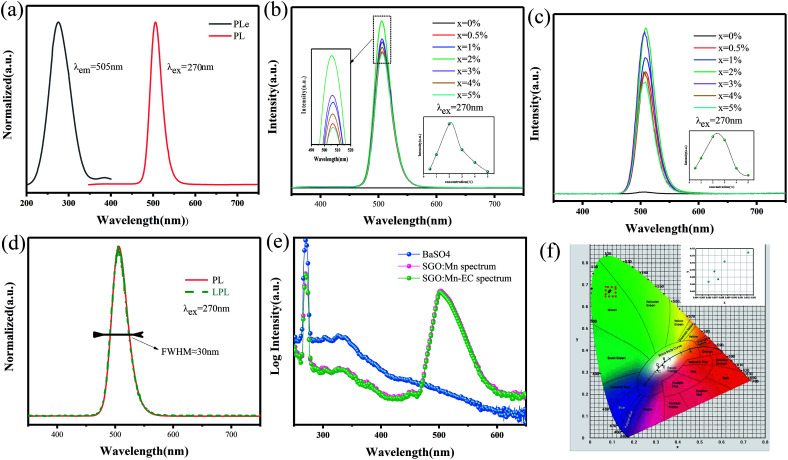
(a) The PL and PLE spectra of SGO:1% Mn^2+^; (b) the PL spectra of SGO:*x*Mn^2+^; (c) the LPL spectra of SGO:*x*Mn^2+^; (d) the normalized PL and PLE spectra of SGO:2% Mn^2+^; (e) the IQE of SGO:Mn phosphor and SGO:Mn–EC powder; (f) the CIE 1931 color coordinates of SGO:*x*Mn^2+^ (*x* = 0%, 0.5%, 1%, 2%, 3%, 4%, and 5%).

A suitable emission wavelength and a certain luminous intensity are necessary for a visual fluorescent probe. The PL spectra and PLE spectra of un-functionalized SGO:1% Mn^2+^ are shown in Fig. 3(a). The excitation band from 200 to 400 nm may result from the charge-transfer transition or the d^5^–d_*s*_^4^ transition of Mn^2+^ ions, while the 505 nm emission can be attributed to the ^4^T_1_–^6^A_1_ transition in Mn^2+^.^39^ A green emission is expected due to Mn^2+^ ions at the tetrahedral coordination sites with a weak crystal field.^40,41^ In contrast, for Mn^2+^ ions at octahedral coordination sites with a strong crystal field yield, a longer wavelength range from orange to red emission should be observed.^42,43^ Herein, Mn^2+^ ions certainly occupied tetrahedral coordinated Ga^3+^ sites surrounded by four oxygen ions. Mn^2+^ emission occurs as a narrow band due to weak electric-phonon interactions and low structural relaxation caused by the highly-rigid crystal structure and symmetric coordination environment.^44^ The PL spectra of SGO:*x*Mn^2+^ (*x* = 0%, 0.5%, 1%, 2%, 3%, 4%, and 5%) are shown in Fig. 3(b). The intensity of the emission peaks increased with the Mn^2+^ concentration, reached a maximum at 2%, and finally decreased due to concentration quenching. The relationship between intensity and concentration is shown in the inset.

The LPL spectrum in Fig. 3(c) shows that the relationship between intensity and concentration of the LPL spectrum is the same as that in the PL spectrum. The normalized PL and LPL spectra are shown in Fig. 3(d), which indicated that PL and LPL shared the same luminescence centers because both the peak shape and peak position are identical. The full width half maximum (FWHM) is very narrow (close to 30 nm), which is suitable for backlighting displays.^44–46^ The peak position is 505 nm, which is the most sensitive band of the human eye, making materials easier to identify in the dark.^47^ These luminescence properties can potentially be used in visualization display applications.

The LPL can cause, the release of electrons or holes from traps under certain thermal excitation and information about the traps can be obtained from thermo-luminescence (TL) spectra. Fig. S2(a)† shows the TL curves for the SGO:*x*Mn^2+^ phosphor. The SGO itself has many traps and doping it with Mn^2+^ increases the trap density, as shown in Fig. S2(b),† making the SGO:Mn phosphor particularly suitable for gated fluorescence trap applications. The persistent luminescence of SGO:Mn persisted for more than 3000 s, which makes is suitable for efficient time-gated LFPs visualization, as shown in Fig. S3.† The insets of Fig. S3† show the digital photograph of visualization of the SGO:Mn–EC powder at different decay time (0 s, 30 s, 60 s, 120 s, and 300 s); the photograph should be taken before 300 s in order to get a clear image of the fingerprint. Fig. 3(e) shows the IQE of SGO:Mn phosphor and SGO:Mn–EC powder; the IQE decreased slightly from 46.66% to 43.61% after the attachment of EC.

Generally speaking, a narrower FWHM usually implies higher color purity, which is used to express the brightness and depth of a color, and a high color purity exhibits a high contrast against an ambient environment.^14,45^ The CIE diagrams of SGO:*x*Mn^2+^ (*x* = 0%, 0.5%, 1%, 2%, 3%, 4%, and 5%) are shown in Fig. 3(f). The color purity of a specific color is defined as the percentage of the linear distance between the chromaticity coordinates of the measured plot and the white light source to the linear distance between the chromaticity coordinates of the monochromatic light source and the white light source. It can be calculated by the following equation:1
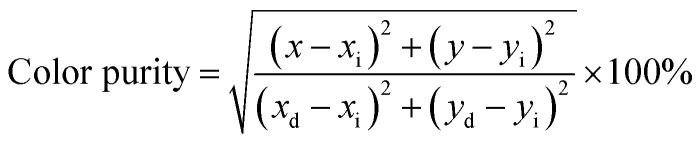
where (*x*, *y*) represents the CIE color coordinates of the SGO:2% Mn^2+^ phosphors, (*x*_i_, *y*_i_) represents the white light source with CIE color coordinates (0.3333, 0.3333), and (*x*_d_, *y*_d_) are the color coordinates corresponding to the monochromatic light source.^14^ The color purity of the SGO:2% Mn^2+^ phosphor was calculated to be 81.3%, which is higher than the commercial green phosphor β-SiAlON:Eu^2+^.^48^ This shows that this phosphor is suitable for displays. The color purities of different Mn^2+^ concentrations are shown in Table S4.†

**Fig. 4:**
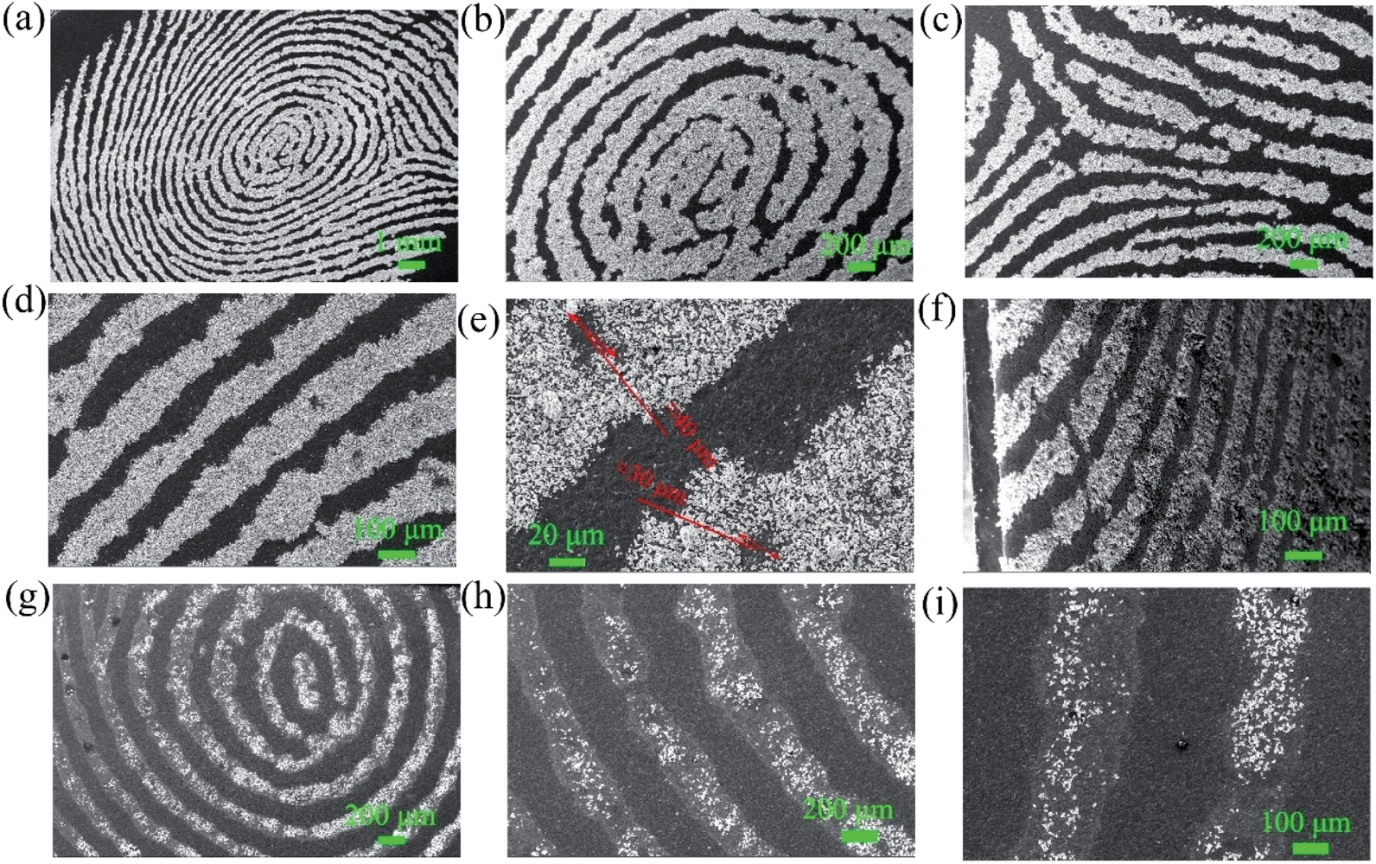
(a–f) The SEM imaging of LFPs on a silicon wafer substrate covered with SGO:Mn–EC. (a) Full view of LFPs; (b–e) sectional view of LFPs; (f) the cutaway of the silicon wafer substrate covered with SGO:Mn–EC. (g–i) The SEM imaging of LFPs on the silicon wafer substrate covered with the SGO:Mn phosphor alone. (g) Full view of the LFPs; (h and i) sectional view of the LFPs.

When fingers touch surfaces covered in substances such as sweat, grease, or other contaminants, an impression of the fingerprint's ridges and furrows is left behind. When applied to different substrates containing LFPs, powder mixtures become immobilized along the ridges of the fingerprints. SGO:Mn–EC was attached to the surface of the substrate by powder dusting and the hydrophobic interaction increased the strength of their connection. Fig. 4 shows the SEM images of LFPs on a silicon wafer substrate covered with SGO:Mn–EC. The primary and secondary features of LFPs were clear enough to determine significant information of the identity characteristics, and the ridge and groove details can be obviously observed at the 1 mm scale in Fig. 4(a). The first level features of the fingerprint core are shown in Fig. 4(b) and the second level features of the fingerprint delta are shown in Fig. 4(c) at the 500 μm scale. By increasing the SEM magnification, additional fingerprint details can be observed, including pores (generally 20–50 μm) on the fingerprint ridges in Fig. 4(d). On further increasing the SEM magnification in Fig. 4(e), no distribution of the powder particles in the grooves was seen, the pores marked were 30 and 40 μm. This provided great selectivity, which refers to the specific effect of the visualization reagent and the fingerprint residue. LFP probes with good selectivity typically adsorb, stain, or chemically react with only the papillary ridge but not with background objects. The third level of fingerprint cores was perfectly resolved due to the excellent selectivity. The ridges and grooves can be better observed in a sectional view of the LFPs in the cutaway in Fig. 4(f). Granules distributed in the ridges were also observed. Fig. 4(g–i) show the SEM imaging of LFPs on silicon wafer substrate covered with SGO:Mn phosphor alone; the SGO:Mn phosphor is sparsely spread on the surface of the fingerprint, which indicated the poor adhesion of inorganic phosphor alone. Only the level 1 information of the fingerprint can be observed.

**Fig. 5:**
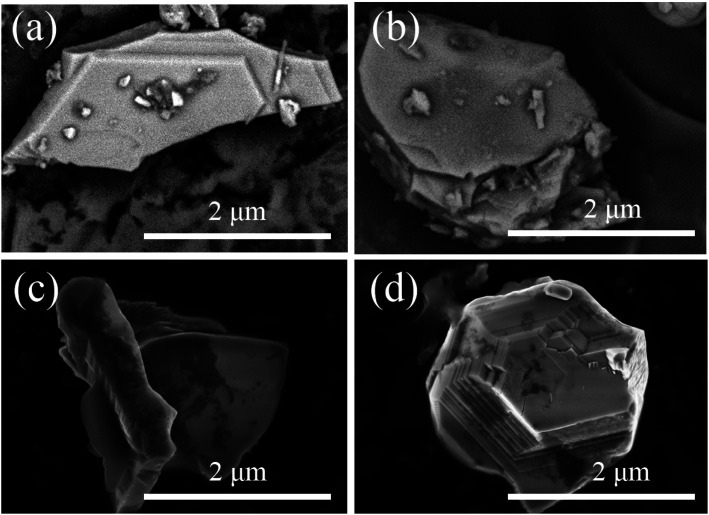
The morphology of SGO:Mn–EC. (a and b) The morphology of SGO:Mn; (c and d) the morphology of functionalized SGO:Mn–EC.

The particle surface topography changed after the SGO:Mn phosphor was functionalized with EC. By comparing Fig. 5(a and b), EC distributed on the surface of SGO:Mn caused a black shadow, as observed in Fig. 5(c and d), while clean surfaces were observed in Fig. 2(a). This shows that EC was attached to the phosphor surface through a simple functionalization.

**Fig. 6:**
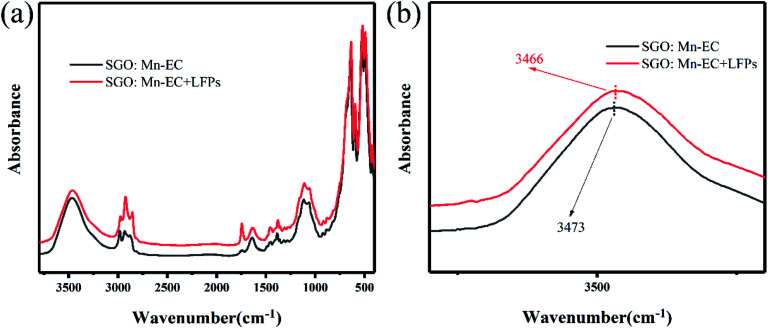
FTIR spectra (a) *λ* = 3800–400 cm^−1^; (b) *λ* = 3600–2000 cm^−1^ of SGO:Mn–EC and SGO:Mn–EC combined with LFPs.

To confirm the hydrophobic interaction between the SGO:Mn–EC powder and LFPs, a series of infrared absorption spectra was obtained. An absorption band was observed from 500 to 600 cm^−1^ for six-coordinated Ga^3+^ and from 600 to 700 cm^−1^ for four-coordinated Ga^3+^.^49^ The Sr–O band was not observed due to Sr–O stretching and O–Sr–O bending vibrations from 150–250 and 100–140 cm^−1^.^50^ There was no peak shift observed in pure SGO:Mn compared with SGO:Mn on the LFPs. After adding the LFPs by finger touching the surface of the KBr pellet, some peaks appeared due to the organic matter at 2923 cm^−1^ and 1740 cm^−1^.^22^ In pure EC, the water peak shifted from 3472 cm^−1^ to 3465 cm^−1^ in Fig. S4.† A peak shift was also observed in the organic–inorganic hybrid SGO:Mn–EC powder (Fig. 6), where the peak at 3469 cm^−1^ shifted to 3465 cm^−1^, indicating the presence of hydrophobic interactions.^22^ Such chemical adsorption is stronger than physical adsorption, which increased the strength of connections between the powder and the fingerprint, thereby increasing the resolution of the resolved fingerprints. The main peaks of EC are listed in Table 2.

**Table 2:** Wavenumbers of the main peaks in the FTIR spectrum of EC (cm^−1^)

Wavenumber (cm^−1^)	3472	2976	2929	1639	1445	1379
	–OH	–CH	–OH	C–O	–CH_2_	–CH_3_

**Fig. 7:**
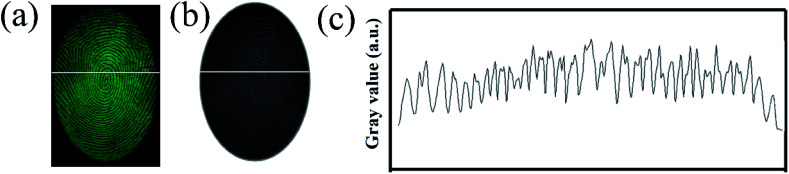
(a) The digital photographs; (b) the grayscale photos; (c) the gray value over the LFPs indicated by the SGO:Mn–EC powder.

In order to verify the performance in practical applications, all the SGO:Mn–EC powders were applied to different surfaces using the powder method. After a few seconds of irradiation with a 254 nm UV lamp, the excitation source was immediately removed. Then, the images of the LFPs on smooth glass and rough wood surfaces were obtained by photoluminescence and time-gated persistent luminescence, as shown in Fig. S5.† The SGO:Mn–EC powder has better resolution than commercial black powder. The LFPs form different donors can be clearly visualized, as shown in Fig. S6.† Fig. S6(a–f)† corresponded to the donors varying with age and gender, which indicated that the universality of LFP visualization varies with the donors. Fig. 7(a) shows the digital photograph of fingerprint visualization using the SGO:Mn–EC powder; the grayscale photos of Fig. 7(a) are transformed, as shown in Fig. 7(b). Fig. 7(c) shows the grayscale variation imaged by the solid white line in Fig. 7(a and b). The images of the fingerprint ridges and grooves obtained by SGO:Mn–EC have a significantly high contrast.

**Fig. 8:**
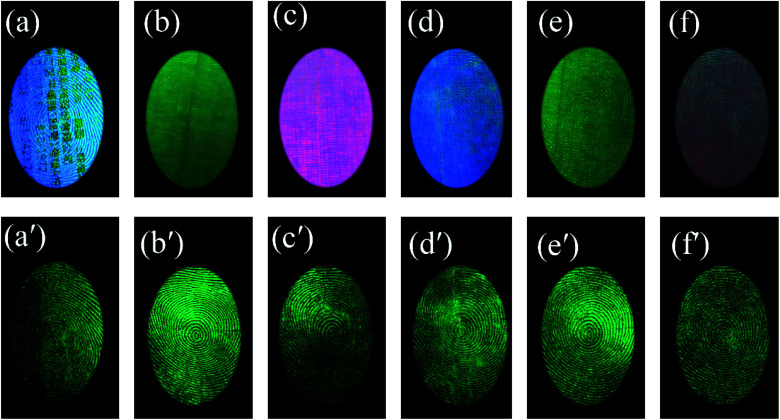
(a–f) A series of acrylic sheets with different fluorescence colors; photoluminescence imaging of LFPs on different substrates with strong fluorescence; (a′–f′) the time-gated LFP imaging. (a and a′) The printer paper with a word, (b and b′) green acrylic sheet, (c and c′) pink acrylic sheet, (d and d′) blue acrylic sheet, (e and e′) olivine acrylic sheet, and (f and f′) red acrylic sheet.

The powder also showed a rapid response to ultraviolet excitation and a high afterglow intensity. After a few seconds of irradiation, the excitation light source can be removed for photographing, which prevents damage to the eyes of the observer with ultraviolet light for an extended time. Fingerprint visualization *via* time-gated florescence can prevent all background interference, which allows a high-resolution image to be obtained (Fig. 8). There are 6 kinds of substrates with strong background interference, as shown in Fig. 8(a): a series of acrylic sheets with different fluorescence colors, *viz.*, green, blue, pink, and olivine from Fig. 8(b–f). Under the influence of strong background fluorescence, the fingerprint image becomes unclear and is concealed, and the details cannot be observed. However, LFP imaging *via* time-gated fluorescence still showed a very clear shape, whose details including level 1 information and level 2 information of the fingerprints can also be seen in Fig. 8(a′–f′).

**Fig. 9:**
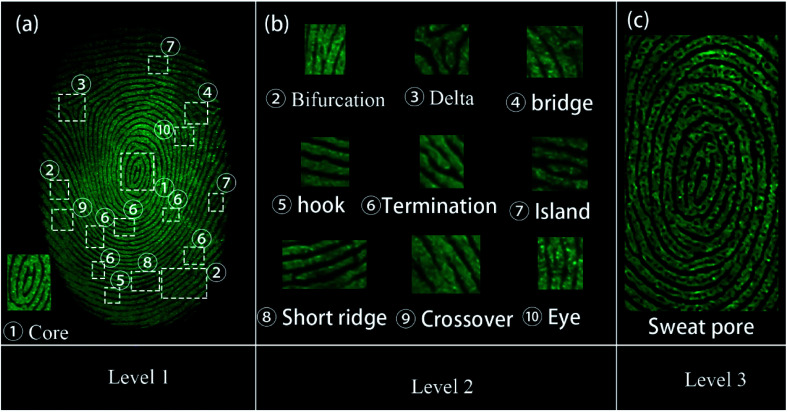
Images of three levels of LFPs on the glass developed through powder method with excitation wavelength of 254 nm. (a) The level 1 of LFPs imaging. (b) The level 2 of LFPs imaging. The magnified images show specific details as listed below, ① – core; level 2, ② – bifurcation, ③ – delta, ④ – bridge, ⑤ – hook, ⑥ – termination, ⑦ – island, ⑧ – short ridge, ⑨ – crossover, and ⑩ – eyes; (c) the level 3 of LFPs imaging.

All the three levels of the LFP features could be obtained using the SGO:Mn–EC powder due to its favorable selectivity. The imaging of the three levels of LFP features on glass developed using the powder method are shown in Fig. 9. Level 1 included the patterns of the fingerprints, which can be recognized from Fig. 9(a). Level 2 included bifurcations, deltas, bridges, hooks, terminations, islands, short ridges, crossovers, and eyes, as shown in Fig. 9(b). Level 3 information was obtained by enlarging the images around the core in Fig. 9(a); the typical sweat pores and contours can been clearly identified, as shown in Fig. 9(c). The level 3 information of fingerprints is too microcosmic to be distinguished by naked-eyes and should be obtained by increasing the magnification of the image. The level 3 features are the most useful and are especially useful for the identification of partial, fuzzy, or deformed fingerprints.^51^ For example, information can be determined from the relative position of the sweat pores in the two fingerprint intersections in Fig. S7.†

**Fig. 10:**
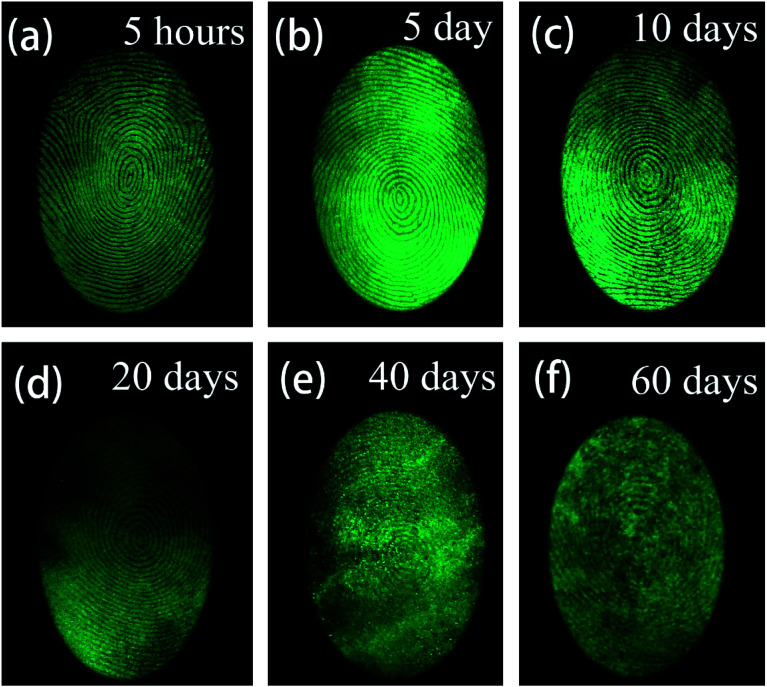
Fluorescent images of LFPs developed by the powder method (a) aged for 5 hours, (b) aged 5 days, (c) aged for 10 days, (d) aged for 20 days, (e) aged for 40 days, (f) aged for 60 days.

During criminal investigations, most fingerprints are not fresh and the detection limit of LFP imaging is interrelated to the visualization of aged ones. For increasing fingerprint age, the substructure of LFPs can still be detected, as shown in Fig. 10. Even at an age of 60 days, the LFP can still be visualized by the SGO:Mn–EC powder, which demonstrates that a low detection limit can be achieved by the powder. It is worth mentioning that heating the fingerprints caused grease to diffuse in the fingerprint, which had an effect similar to the aging process (Fig. S8†). The obtained LFP visualization was blurred by the powder dusting method after heating at 100 °C and longer heating times from 10 min to 30 min increased the blurriness. The powder dusting method was utilized in this work, which avoids damage to the fingerprint by the heating operation.

Chemical stability is also an important parameter in practical applications; the stability of inorganic substances is usually better than that of organic matter.^3^ The surface functionalization of EC enhanced the stability of the powder due to the protection by EC. The powder was placed in different chemical environments, including an acetic acid solution for an acidic environment (pH = 4.0), a neutral environment (pH = 6.8), and an ammonia solution for an alkaline environment (pH = 10.0), as shown in Fig. S9.† Both the photoluminescence and afterglow were nearly unaffected for up to 30 days. An accelerated experiment was carried out by raising the temperature from room temperature to 200 °C. The photoluminescence and afterglow remained stable below 150 °C but the photoluminescence intensity remained stable at higher temperatures. The afterglow intensity was attenuated as the temperature increased. Overall, this powder showed good chemical stability.

## 4 Conclusion

In this work, a narrow-band emitting green afterglow phosphor, SGO:Mn, functionalized with ethyl cellulose (SGO:Mn–EC) was utilized as a fluorescence probe for the visualization of LFPs. The organic–inorganic hybrid powder overcome many problems often seen in fluorescent probes used for LFPs visualization. The favorable hydrophobic interactions occurring between the hydrophobic groups in ethyl cellulose and the lipid-rich LFPs more strongly adhered to the ridges improved the selectivity of LFPs recognition. Monochromatic green time-gated emitting improved the contrast of LFPs recognition. Background fluorescence of the substrates was completely avoided because of the luminescence of the afterglow phosphor. All three levels of LFP features were clearly observed. Such an organic–inorganic hybrid strategy provides a technically simple, chemically stable, low-toxic, low-cost, rapid, and easy method to obtain high-contrast visualization of LFPs. This type of strategy may have broad application prospects in criminal investigations.

## Conflicts of interest

There are no conflicts to declare.

## Supplementary Material

RA-010-D0RA00138D-s001
