# Supplementary material for: Novel organic–inorganic hybrid powder SrGa12O19:Mn2+–ethyl cellulose for efficient latent fingerprint recognition via time-gated fluorescence
Source: RSC Adv. 2020 Feb 26;10(14):8233–43. doi: 10.1039/d0ra00138d (PMC9049934; doi:10.1039/d0ra00138d)
Supplement: RA-010-D0RA00138D-s001 [file RA-010-D0RA00138D-s001.pdf]

Electronic Supplementary Material (ESI) for RSC Advances.
This journal is © The Royal Society of Chemistry 2020

Electronic Supplementary Information

Novel Organic-Inorganic Hybrid powder $SrGa_{12}O_{19}$:Mn^{2+}-Ethyl Cellulose for Efficiency Latent Fingerprint Recognition via Time-Gated Fluorescence

Jun'an Lai [1], Zhangwen Long [1], Jianbei Qiu [1, 2]*, Dacheng Zhou [1, 2], Qi Wang [1, 2]*, Yong Yang[1], Songhan Hu [1], Zhe Wang [1] , Ke Zhang [1],

[1] College of Materials Science and Engineering, Kunming University of Science and Technology, Kunming 650093, China

[2] Key Lab. of Advanced Materials of Yunnan Province, Kunming 650093, China

*Corresponding author: Jianbei Qiu, E-mail address: qiu@kmust.edu.cn

Qi Wang, E-mail address: 363270387@qq.com

Tel:+86-871-5188856; Fax: +86-875-5188856

Table S1 The Wyckoff position, atomic coordinates and the occupancies of SGO: 2%Mn(A)

Site	Wyckoff position	x	y	z	occupancy
Sr1	2d	0.3333	0.6667	0.75	1
Ga1	2a	0	0	0	1
Ga2	4e	0	0	0.2574	1
Ga3	4f	0.3333	0.6667	0.02686	1
Ga4	4f	0.3333	0.6667	0.18992	1
Ga5	12k	0.169	0.33692	0.89085	1
O1	4e	0	0	0.14825	1
O2	4f	0.3333	0.6667	0.94681	1
O3	6h	0.1842	0.36729	0.25	1
O4	12k	0.1569	0.31287	0.05212	1

O5	12k	0.5055	0.00999	0.15081	1

Table S2 The Wyckoff position, atomic coordinates and the occupancies of SGO: 2%Mn(B)

Site	Wyckoff position	x	y	z	occupancy
Sr1	2d	0.3333	0.6667	0.75	1
Ga1	2a	0	0	0	1
Ga2	4e	0	0	0.25719	1
Ga3	4f	0.3333	0.6667	0.02692	1
Ga4	4f	0.3333	0.6667	0.18992	1
Ga5	12k	0.16896	0.33692	0.89084	1
O1	4e	0	0	0.14809	1
O2	4f	0.3333	0.6667	0.94666	1
O3	6h	0.18415	0.36729	0.25	1
O4	12k	0.15692	0.31287	0.05211	1
O5	12k	0.50547	0.00999	0.15073	1

Table S3 The mass and atomic percent of each element in SGO: 5 % Mn by SEM-EDS analysis

Element	Weight %	Atomic %	Net Int.	Error %	Kratio	Z	R	A	F
O K	32.84	68.49	324.7	8.39	0.09	1.18	0.88	0.24	1.00
MnK	0.75	0.46	32.7	3.33	0.01	0.94	1.02	0.96	1.12
GaK	58.93	28.20	1190.1	1.10	0.54	0.90	1.05	1.01	1.01
SrK	7.47	2.85	25.2	4.90	0.06	0.84	1.06	0.97	1.00

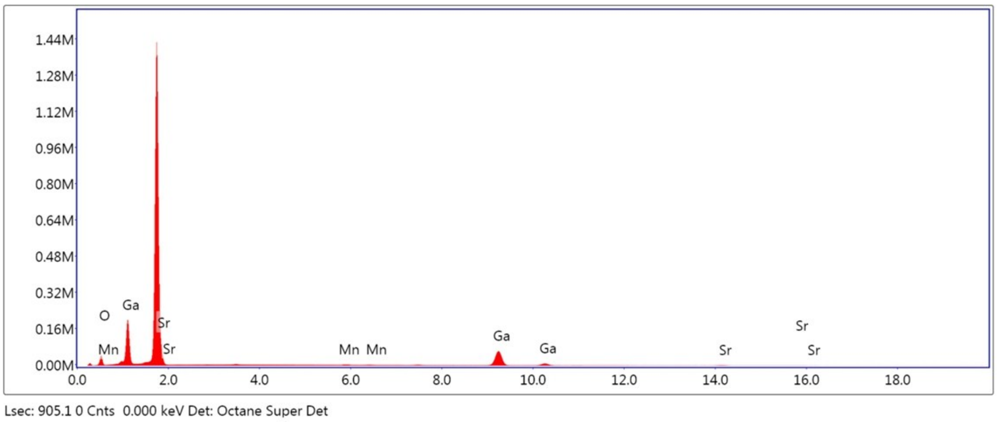

Fig. S1 The SEM-EDS analysis of SGO: 5% Mn.

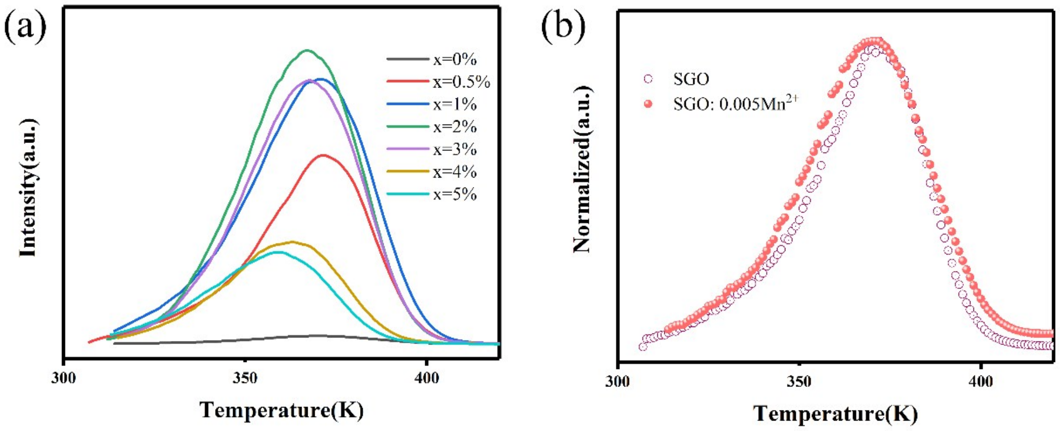

Fig. S2 (a) The TL spectra of SGO: xMn^{2+} (x =0%, 0.5%,1%, 2%,3%,4%,5%); (b) The normalized TL spectra of SGO: 0.5%Mn^{2+} and un-doped SGO.

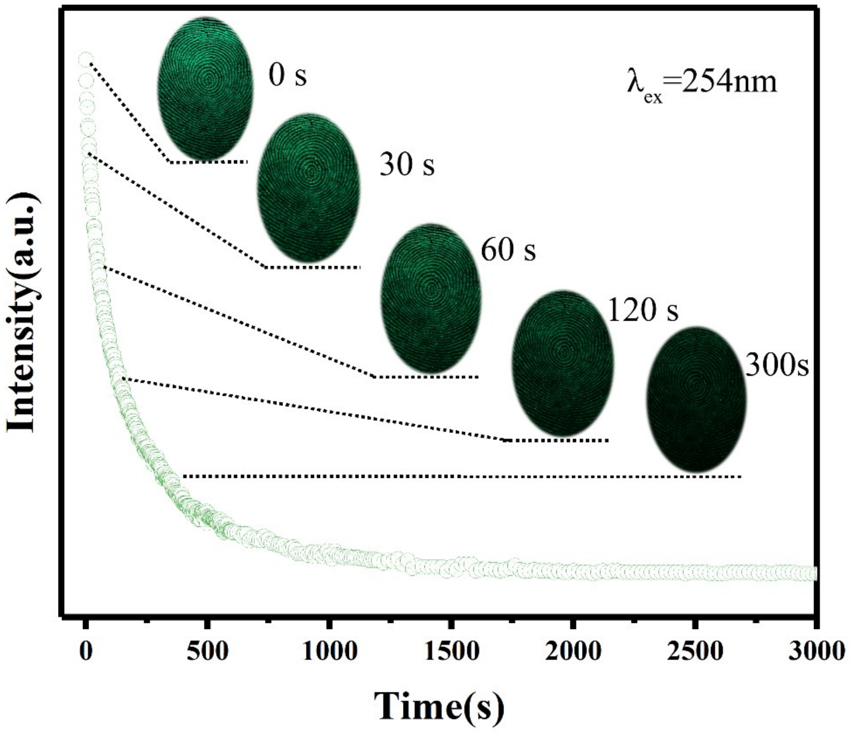

Fig. S3 The time-gated LFPs visualization by SGO: Mn-EC powder.

Table S4 The color purities of SGO: xMn2+ (x =0.5%, 1%, 2%, 3%, 4%, 5%)

concentration	0.5%%	1%,	2%	3%	4%	5%
color purity	80.61%	84.75%	81.28%	80.69%	83.9%	81.6%

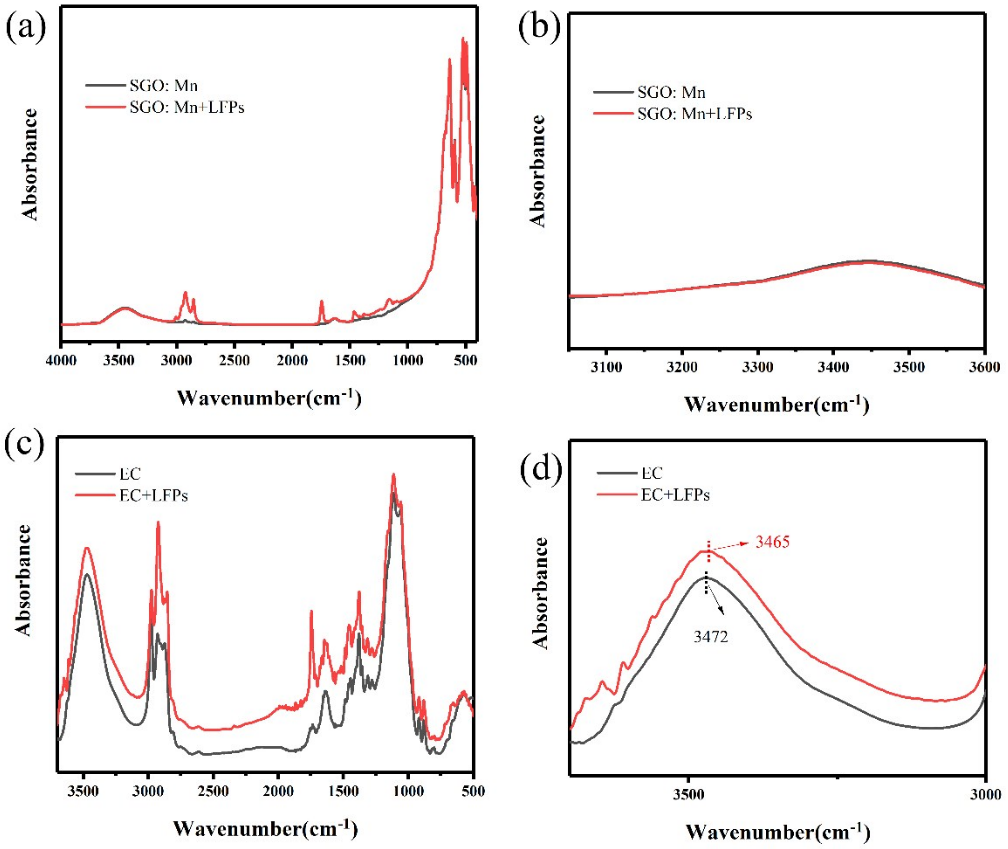

Fig. S4 FTIR spectra (a) (b) FTIR spectra of SGO: Mn and SGO: Mn combined with LFPs; (c) (d) FTIR spectra of EC and EC combined with LFPs.

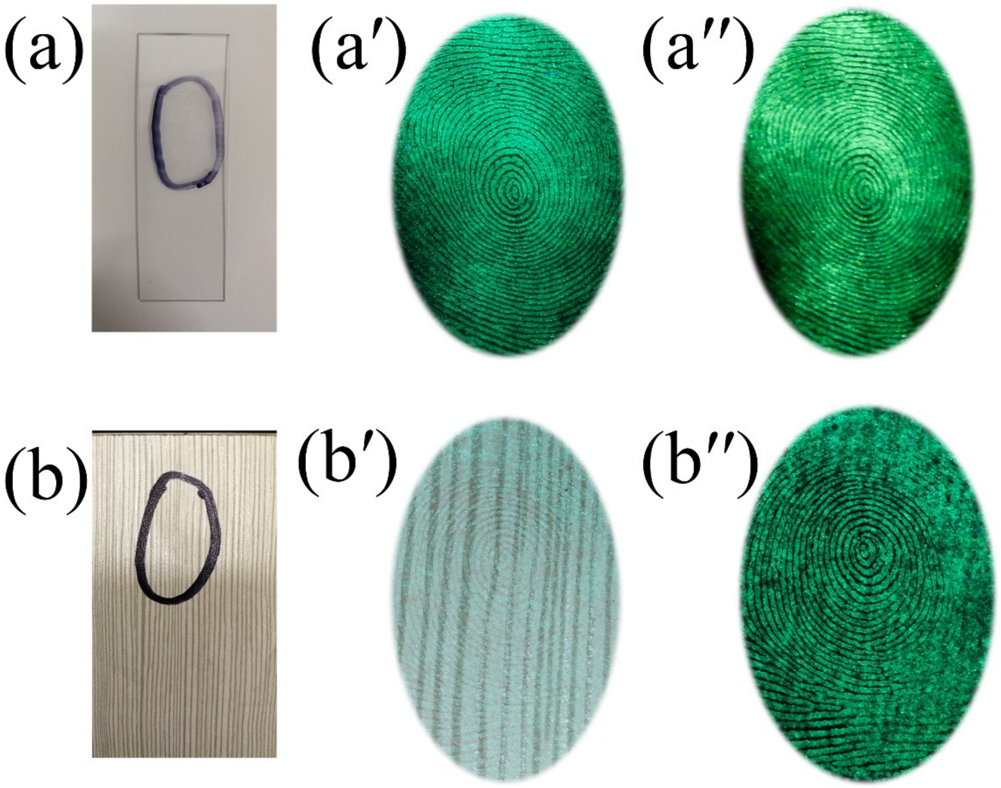

Fig. S5 (a) Fingerprint recognition on smooth glass surface; (b)fingerprint recognition on rough wood surface.

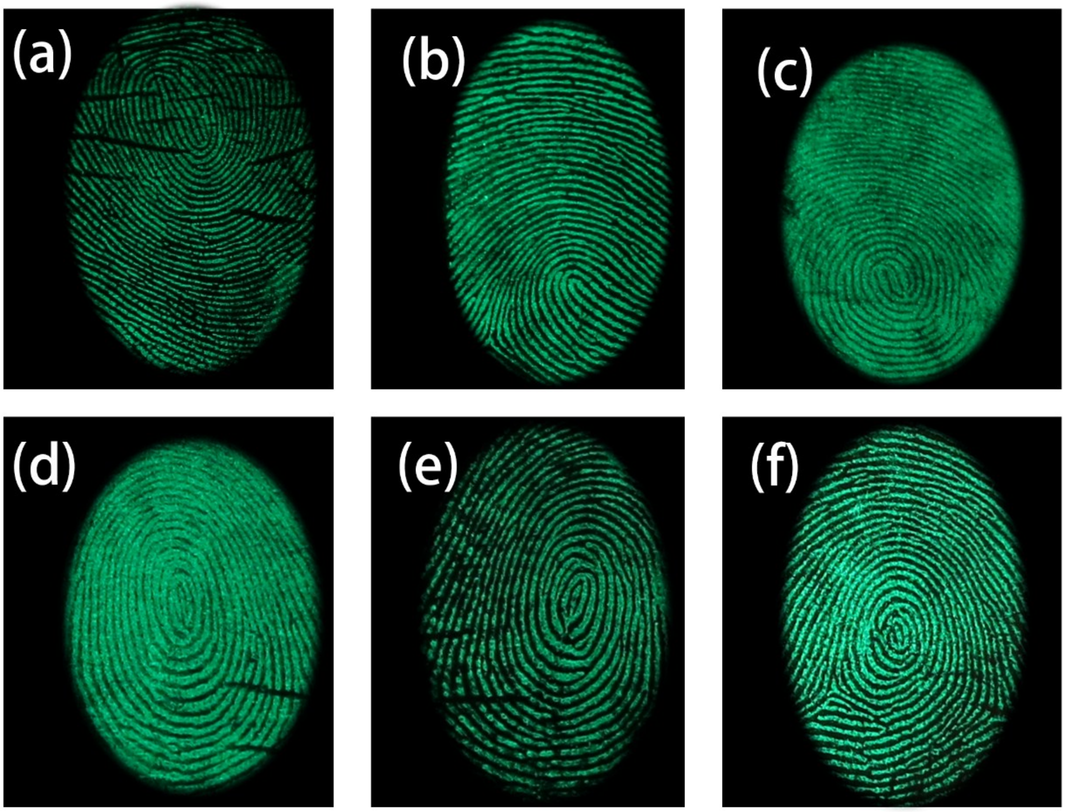

Fig. S6 The visualization of LFPs from different donors. (a) 24 years old male; (b) 28 years old male; (c) 24 years old female; (d) 30 years old male; (e) 26 years old female; (f) 24 years old male.

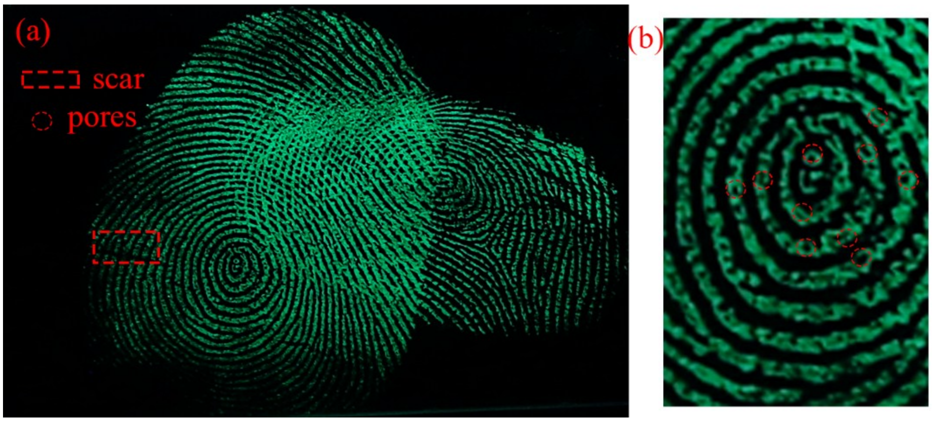

Fig. S7 (a)The fingerprint recognition of cross-fingerprints, (b) The typical pores by partial magnification of the cross-fingerprints.

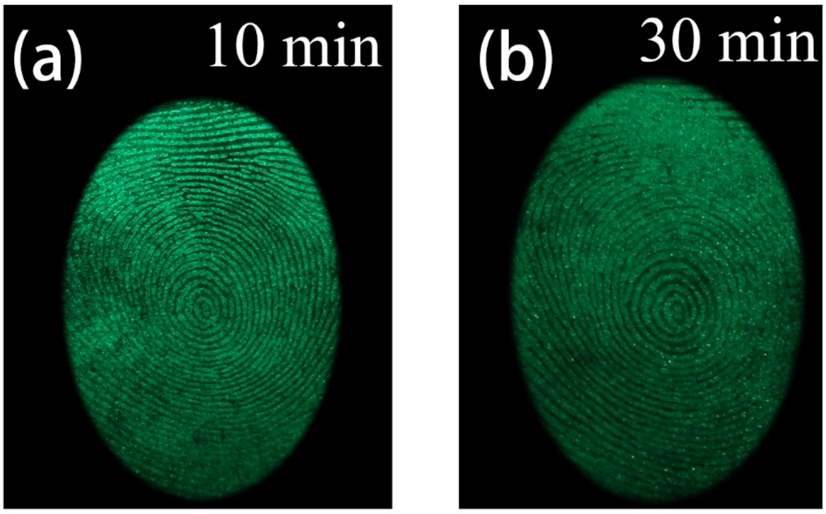

Fig. S8 Fingerprint recognition after heating at 100 °C for different time, (a)10 min; (b)30 min.

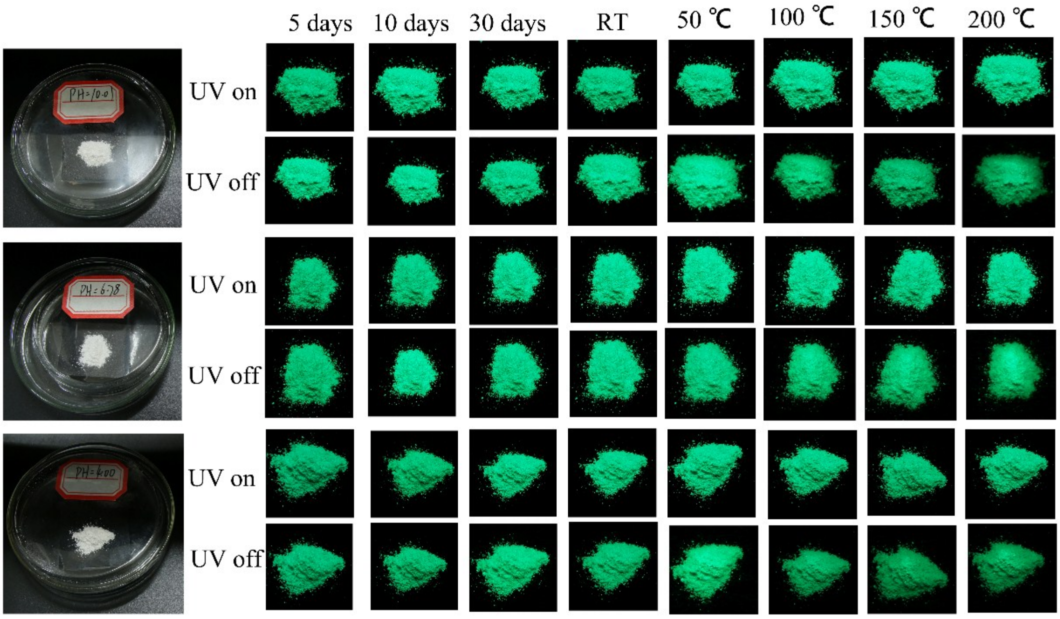

Fig. S9 Weather resistance of SGO: Mn-EC powder.
